# Eardrum-inspired soft viscoelastic diaphragms for CNN-based speech recognition with audio visualization images

**DOI:** 10.1038/s41598-023-33755-2

**Published:** 2023-04-19

**Authors:** Seok-Jin Park, Hee-Beom Lee, Gi-Woo Kim

**Affiliations:** Department of Mechanical Engineering, Inha University, 100 Inha-ro, Michuhol-gu, Incheon, 22212 Republic of Korea

**Keywords:** Soft materials, Electrical and electronic engineering

## Abstract

In this study, we present initial efforts for a new speech recognition approach aimed at producing different input images for convolutional neural network (CNN)-based speech recognition. We explored the potential of the tympanic membrane (eardrum)-inspired viscoelastic membrane-type diaphragms to deliver audio visualization images using a cross-recurrence plot (CRP). These images were formed by the two phase-shifted vibration responses of viscoelastic diaphragms. We expect this technique to replace the fast Fourier transform (FFT) spectrum currently used for speech recognition. Herein, we report that the new creation method of color images enabled by combining two phase-shifted vibration responses of viscoelastic diaphragms with CRP shows a lower computation burden and a promising potential alternative way to STFT (conventional spectrogram) when the image resolution (pixel size) is below critical resolution.

## Introduction

Speech recognition, also known as speech-to-text (STT), is defined as a process that enables a program to recognize and translate human speech (or voice) into a written format. The associated methods provide one of the most intuitive human–machine interfaces (HMI)^1,2^. Speech recognition methods enable voice user interface systems to replace or supplement conventional touch-based HMI devices. Accordingly, they have attracted considerable attention as a core technology of artificial intelligence assistants and the internet of things associated with smart home automation, owing to their interactive convenience and bilateral communication^3–5^. Typically, speech recognition systems involve two main components: an acoustic sensory system and speech recognition software. The acoustic sensor responds to the sound pressure of human speech and converts acoustic energy into an analog electrical signal^6^. The most common acoustic sensors are condenser-type microphones, which use the difference in capacitance between two conducting diaphragms. The responses typically comprise time-series data, which are transformed into the frequency domain using a fast Fourier transform (FFT). In practice, short-time Fourier transform (STFT) plots the modified spectra as a function of time by dividing a longer time signal into shorter segments, which is called a spectrogram. A feature extraction process and an acoustic model are required to implement the speech recognition system, and deep learning algorithms are often employed to improve speech recognition performance under conditions with significant noise.

**Figure 1:**
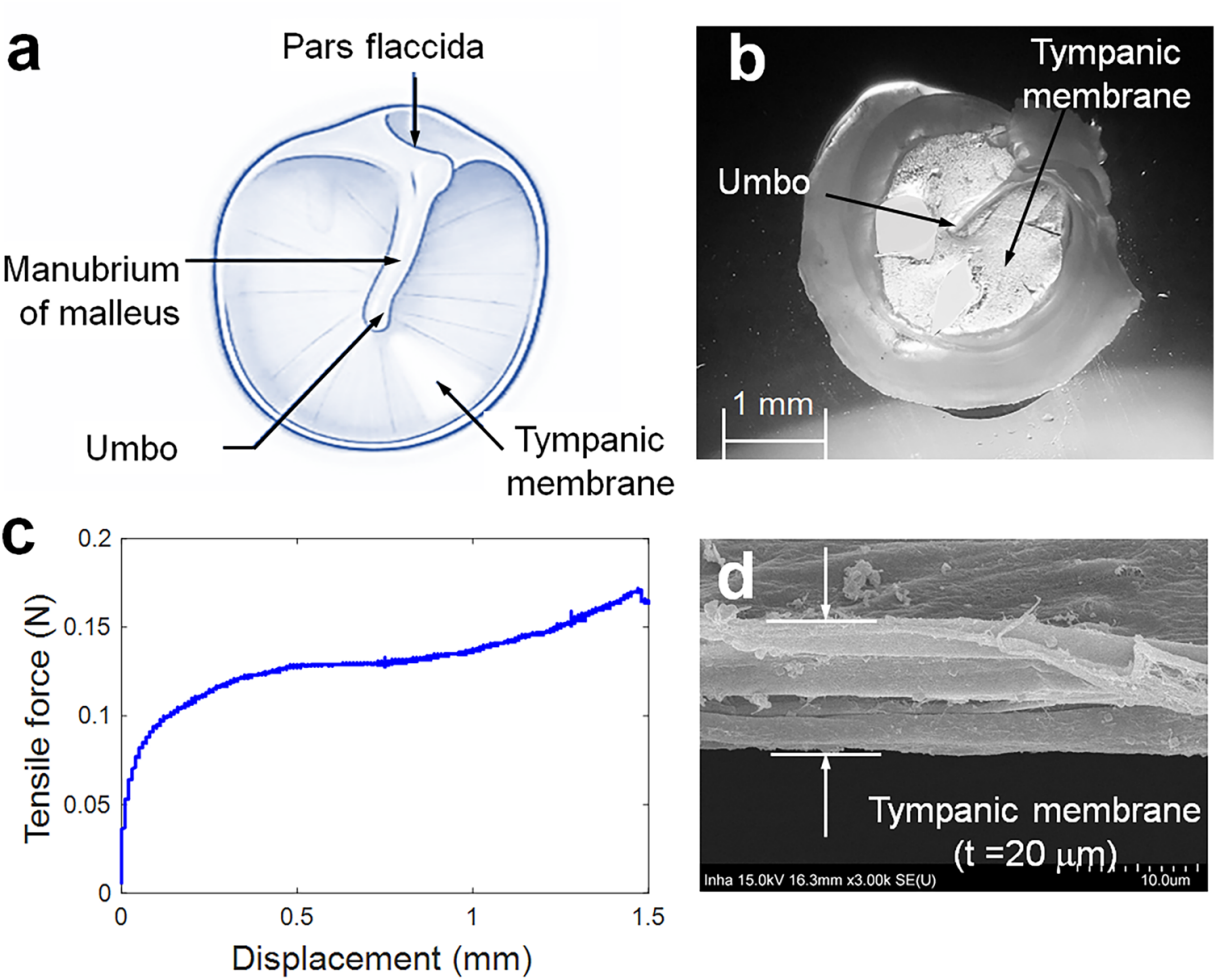
Tympanic membrane (eardrum) as an acoustic diaphragm: (**a**) peripheral view (human), (**b**) microphotography (young rat, provided by Inha University Hospital), (**c**) measured force (stress) vs displacement (strain) curve of a TM extracted from a young guinea pig using a micro tensile tester (Daeyeong), (**d**) cross-sectional microscopic scanning electron microscopy images (young rat, × 100).

To improve the performance of speech recognition, significant studies have focused on the development of improved acoustic sensors. Previous studies have considered various types of acoustic sensors, including triboelectric, piezoelectric, piezocapacitive, and piezoresistive sensors^7^, and devoted efforts to enhance their sensitivity and frequency response function (ideally to uniform). Recent studies have reported various flexible skin sensors that can detect human voices by analyzing the vibrations in the vocal cords^7^. Significant efforts have been made to develop new acoustic sensors (microphones)^8^. Because the tympanic membrane (TM, also known as the eardrum) plays a key role in transmitting acoustic vibrations to the inner ear, acoustic sensors made of viscoelastic polymers have been developed by mimicking the material property of the human eardrum^9^. The thin semi-transparent TM is stretched obliquely across the end of the external canal, as shown in Fig. 1. The manubrium of the malleus is firmly attached to the medial surface of the membrane. The TM is connected to a surrounding annulus ligament (muscle), as shown in Fig. 1a,b. The TM does not move as the typical thin membranes (diaphragm) used in acoustic sensors such as microphones owing to two reasons: (i) the pressure force is transmitted only from the end of the malleus (i.e., umbo) when the TM moves in response to the sound pressure, and (ii) the TM of mammals including humans is a typical viscoelastic material because it is a soft fiber structure consisting of multi-thin layers made of collagen, as shown in Fig. 1d^10^. This viscoelasticity also can be readily evaluated using a stress–strain curve showing a plateau region, as shown in Fig. 1c.

Because sound visualization provides an alternative means to extract speech information such as frequency, many researchers have attempted to visualize sound (or voice information) for speech recognition. Cymatics is a classical sound visualization method wherein different patterns are produced in an excitatory medium depending on the geometry of the plate and the frequency (e.g., Chladni patterns). However, this method is not suitable for speech recognition because it is basically a subset of modal vibrational phenomena^11^. Mak et al. demonstrated the first direct visualization of music in the form of ripples in a microfluidic aqueous–aqueous interface with an ultra-low interfacial tension^12^. This shows the possibility of sensing and transmitting vibrations as minuscule as those induced by sound. A similar study attempted to visualize sound by analyzing the interaction between the acoustic waves and a liquid membrane (soap film)^13^. However, to the best of our knowledge, no prior studies have extracted frequency information, although sound visualization can be used to enhance the understanding of acoustical behavior such as reflection, diffraction, and interference^14,15^. Furthermore, most sound visualization technologies have focused on the localization of sound sources^16,17^. Meanwhile, many researchers attempted to mimic the passive frequency selectivity of the cochlea, which forms a highly sensitive multi-channel frequency filter^18^. Simple frequency selectivity also has been utilized by mimicking cochlea tonotopy using multiple cantilever-beam arrays^19^.

To date, no technology has been completely adopted to replace the current FFT, although the development of alternatives to FFT has remained a topic of considerable interest. Thus, a simpler and more efficient alternative speech recognition system is still required. Therefore, the objective of this study is to develop a new approach to speech recognition, with a primary focus on new acoustic diaphragm. The proposed viscoelastic diaphragm inspired by the TM produces two phase-shifted viscoelastic responses to a sound wave input and delivers new sound (audio) visualization images using the cross recurrence plot (CRP) formed by two phase-shifted vibration responses of viscoelastic diaphragms.

## Eardrum-inspired soft viscoelastic diaphragm

**Figure 2:**
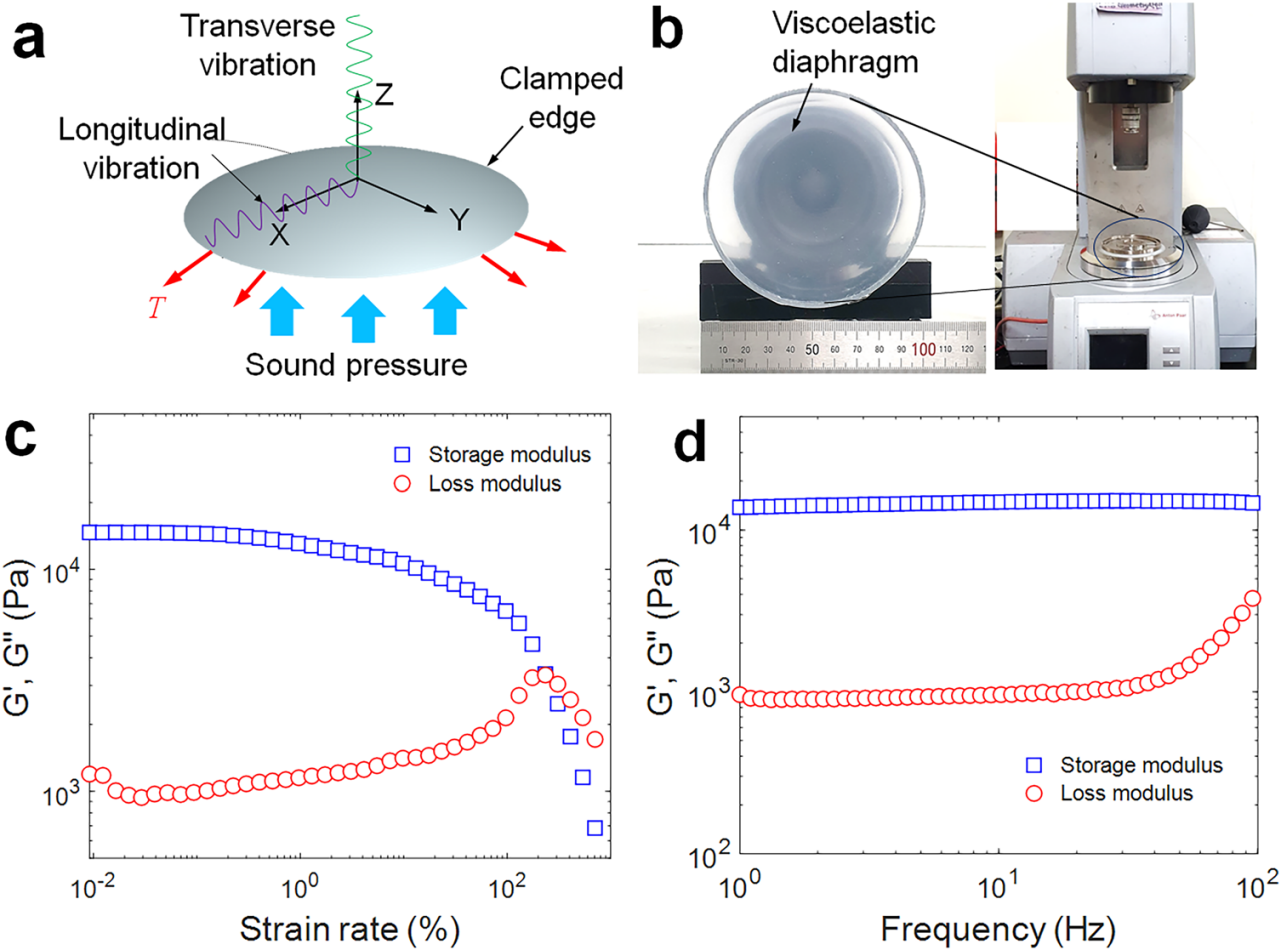
Viscoelastic acoustic diaphragm: (**a**) schematic model, (**b**) photograph, (**c**) storage and loss modulus vs strain rate, (**d**) storage and loss modulus as a function of frequency.

An eardrum-inspired thin circular diaphragm (*ϕ* = 100 mm, *t* = 0.1 mm) made of a silicone rubber film (commercially available from Dongjue Silicone Co., Ltd.) was used as an acoustic vibrating diaphragm (a moving part designed to respond to input sound pressure), as shown in Fig. 2. The circular diaphragm with the clamped edge was driven by sound waves to form transverse (forced) vibrations perpendicular to the diaphragm (i.e., in the* z*-direction). This transverse vibration is governed by the following partial differential equation (i.e., wave equation)^20^:

1 $$\nabla^{2} z - \frac{1}{{c^{2} }}\frac{{\partial^{2} z}}{{\partial t^{2} }} = \frac{q}{{c^{2} \sigma }},$$

where $c$ is constant $(\sqrt {{T \mathord{\left/ {\vphantom {T \sigma }} \right. \kern-0pt} \sigma }} )$, $q$ is a given distributed force, $\sigma$ is the surface density, and $T$ is the tension of the membrane. When the diaphragm is subjected to transverse vibration, a certain point deviated from its equilibrium position (i.e., the center) is unbalanced, and the combined force causes the point to be displaced radially away from its equilibrium position. Consequently, the tension of the string itself acts as a restoring force and causes a longitudinal (natural) vibration parallel to the diaphragm (i.e., radial (x–y) direction), as shown in Fig. 2a.

Because the dynamic responses of viscoelastic materials are generally characterized on the basis of shear modulus (storage and loss modulus), two moduli were measured in an amplitude and oscillation (frequency) sweep test using a rheometer (model: Anton Paar MCR 302, frequency range: 0–100 Hz), as shown in Fig. 2b. The cross-over point was observed at the approximate strain rate of 200% in the amplitude sweep test as an index of viscoelasticity^21^, as shown in Fig. 2c. The viscoelastic responses to a frequency (oscillation) sweep test are represented by the loss modulus as a function of frequency, as shown in Fig. 2d. From these viscoelastic material properties, the expected phase shift (i.e., phase angle difference) between the transverse and longitudinal vibrations of the diaphragms can be derived.

## In-situ measurement of phase shift

**Figure 3:**
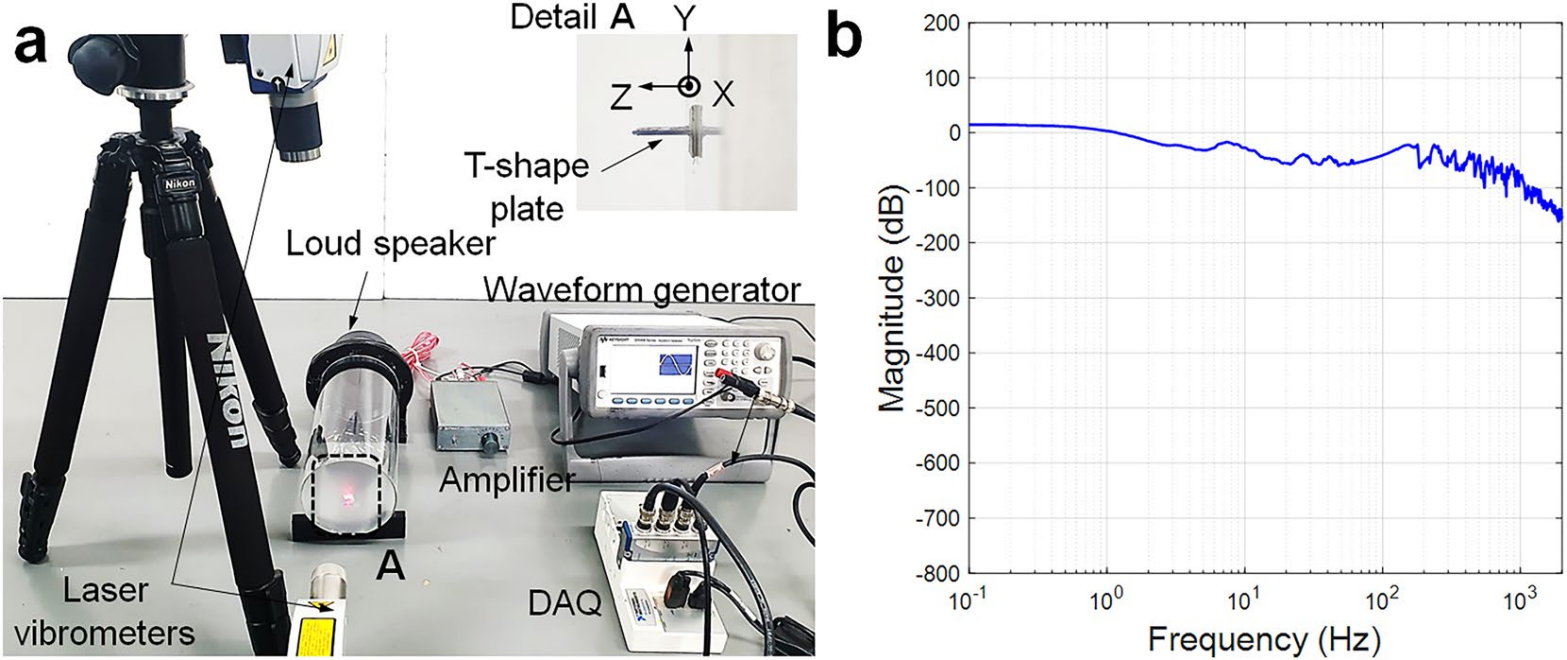
Vibration characteristics of viscoelastic acoustic diaphragm: (**a**) experimental setup, (**b**) its frequency response function (magnitude).

To demonstrate the phase-shifted responses between the transverse and longitudinal vibrations of the diaphragm, two-directional displacements (transverse and longitudinal) were simultaneously measured using two laser Doppler vibrometers (Polytec CLV-2534 and VFX-I-130), as shown in Fig. 3. The steady-state sinusoidal signal from a waveform generator (Keysight, 33500B) was amplified and sent to a loudspeaker to form sound waves, and the diaphragm at the end of the sound guide tube was driven by the sound waves to form transverse vibrations. The T-shape plate with negligible mass was attached to the center of the diaphragm to simultaneously measure the transverse vibration in the *z*-direction and the longitudinal vibration in the *y*-direction. Because most microphones produce their own characteristic frequency responses to sound input over a range of frequencies (typically 20 Hz to 20 kHz), the frequency response function (the ratio of the transverse displacement output to the pressure input; ${{\left| x \right|} \mathord{\left/ {\vphantom {{\left| x \right|} F}} \right. \kern-0pt} F}$) of the diaphragm was recorded, as shown in Fig. 3b. It was nearly uniform over the frequency range of 2 kHz and exhibited considerable damping effects owing to viscoelasticity beyond 2 kHz.

The viscoelastic behavior of the diaphragm causes the two vibration responses to become out-of-phase, and the transverse vibration in the z-direction leads to longitudinal vibration in the *y*-direction with the same frequency as below.

2 $$\, z(t) = \cos (\omega t),\quad y(t) = \cos (\omega t - \phi )$$

**Figure 4:**
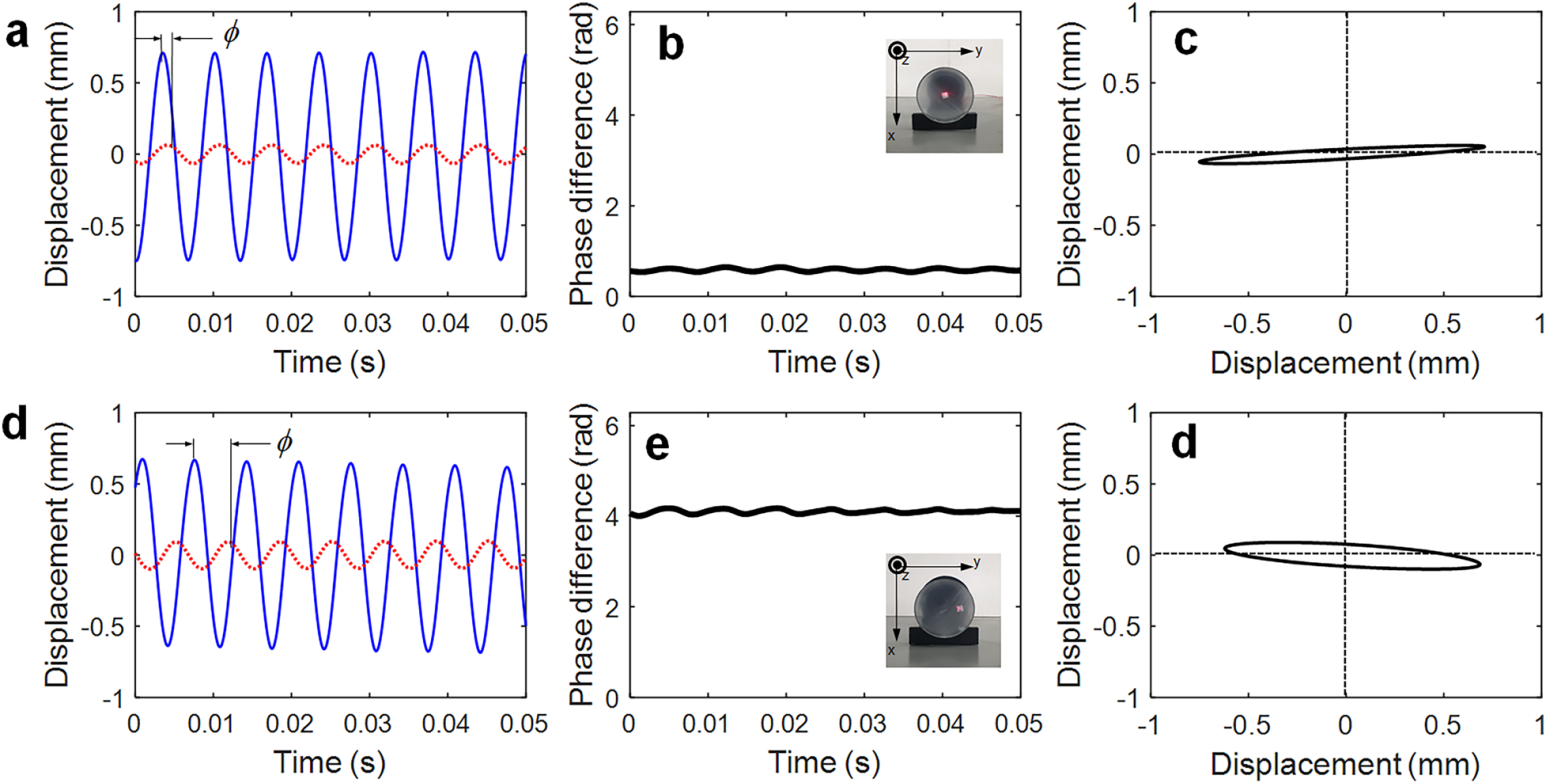
Phase angle difference between the transverse and longitudinal vibrations in the diaphragm at the frequency of 150 Hz: (**a,d**) measured two vibration responses blue line *z*-direction, red dotted line *y*-direction, (**b,e**) phase difference *θ* = 0.59 rad (33.8°) for (**a**) and *θ* = 4.11 rad (235.5°) for (**d**), (**c,f**) its Lissajous curve, (**a–c**) at the center of the diaphragm, (**d–f**) at the off-center of the diaphragm.

Thus, the longitudinal vibration was shifted to the right (i.e., lagged), and a difference in the phase angle (*ϕ*) between the transverse vibration and longitudinal vibrations was clearly observed, as shown in Fig. 4. The Hilbert transform (HT) can be used to calculate instantaneous phase attributes of a time series^22^.

3 $$\, V_{a} (t) = V(t) + i\hat{V}(t) = V(t) + iH[V(t)],$$

where $V(t)$ is the input signal, and $\hat{V}(t)$ denotes the analytic function. The HT returns a complex analytic function from the input signal. Following this, the instantaneous amplitude of the signal (frequently referred to as the envelope function) and instantaneous phase is defined as follows:

4 $$\, A_{i} (t) = \sqrt {\left[ {V(t)} \right]^{2} + \left[ {\hat{V}(t)} \right]^{2} } ,\quad \, \phi_{i} (t) = \arctan \frac{{\hat{V}(t)}}{V(t)},$$

where $\hat{V}(t)$ is the imaginary part of the Hilbert-transformed signal of $V(t)$ and a version of the original real part with a 90° phase shift. The phase difference between two signals can be readily calculated from the instantaneous phase calculated by Eq. (4), as shown in Fig. 4a,d. The phase difference increased with the distance from the center of the diaphragm. For instance, the phase difference increased when the position varied from centered (0.59 rad) to off-center (4.11 rad). These phase shifts can also be characterized by plotting the Lissajous curve (figures). When the input (transverse vibration) is sinusoidal and the output (longitudinal vibration) is sinusoidal with the same frequency, different amplitude, and some phase shift, ellipse-shaped Lissajous curves are produced. Typically, the aspect ratio and inclined angle of the resulting ellipse is a function of the phase shift between the input and output signals, as shown in Fig. 4c,f. This phase shift can be confirmed using laser spirograph images, as shown in Fig. S1.

**Figure 5:**
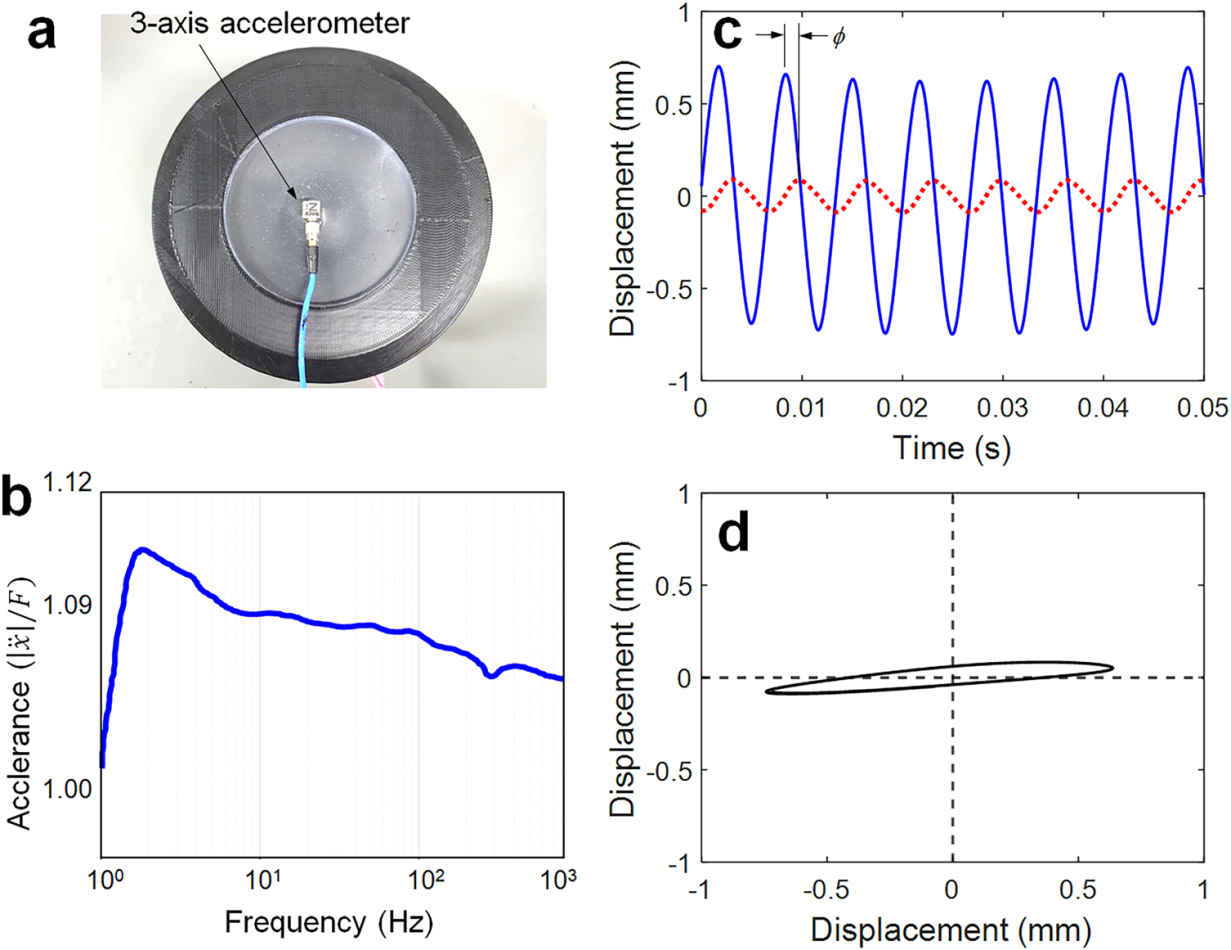
Vibration responses to sinusoidal excitation measured by an in-situ 3-axis accelerometer: (**a**) photograph of 3-axis accelerometer, (**b**) frequency response function (accelerance, ${{\left| {\ddot{x}} \right|} \mathord{\left/ {\vphantom {{\left| {\ddot{x}} \right|} F}} \right. \kern-0pt} F}$), (**c**) two vibration responses to the frequency of 150 Hz: blue line: *z*-direction, red dotted line: *y*-direction, (**d**) its Lissajous curves.

In this study, a three-axis accelerometer (PCB Piezotronics, 356A09) was used for in-situ measurement and characterization (i.e., visualization) of the phase shift between the transverse and longitudinal vibrations because the bulky and expensive laser vibrometer and spirograph were not suitable for speech recognition applications, as shown in Fig. 5a. Inspired by the eardrum (see Fig. 1), this system was used to produce the speech signal from acoustic sound, although an accelerometer is typically ill-suited for measuring frequency response functions in vibration applications. When the compact three-axis accelerometer with a mass of 1 g was attached to the center of the diaphragm, its frequency response function (accelerance, ${{\left| {\ddot{x}} \right|} \mathord{\left/ {\vphantom {{\left| {\ddot{x}} \right|} F}} \right. \kern-0pt} F}$) was a lumped-parameter system similar to the dynamic behavior of the TM. After a resonance of approximately 20 Hz, the function was thus estimated to be slightly rolled off in response to random sound inputs over the frequency range of 1 kHz, as shown in Fig. 5b. For a frequency of 150 Hz shown in Fig. 5c, the calculated phase shift between two vibration responses after double integration of acceleration shown in Fig. 5d was the same as the Lissajous curve in Fig. 4c.

## Application to speech recognition

**Figure 6:**
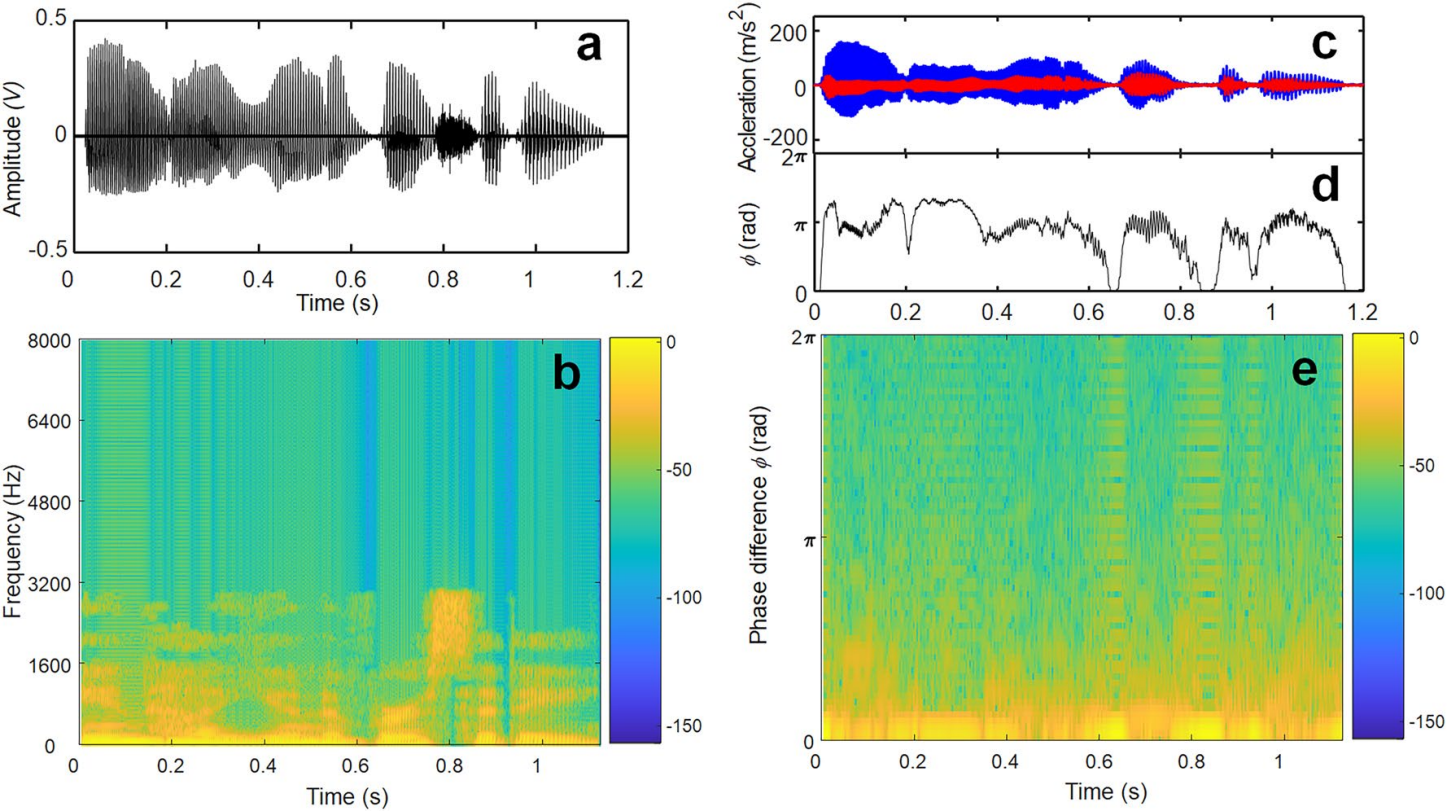
Comparison between STFT and HT (Inha university). (**a**) Speech signal, (**b**) its spectrogram, (**c**) two acceleration signals, (**d**) their phase difference, (**e**) corresponding new spectrogram (time vs. phase difference obtained by HT).

In conventional speech recognition, time-series data should be first transformed into the frequency domain represented by 2D images called a spectrogram, as shown in Fig. 6a,b. Similar to the spectrogram, a new 2D visualized image using HT shown in Fig. 6e is plotted by using two phase-shifted vibration responses (Fig. 6c) and their phase difference (Fig. 6d). The format is a graph with two dimensions: x-axis represents time, and the y-axis represents phase difference whereas y-axis represents is the frequency in the conventional spectrogram. The new visualized image plotted by phase-shifted two signals is obviously similar to the conventional spectrogram (i.e., Fig. 6b), and silent intervals corresponding to no audio signals are also observed in the new spectrogram with HT, which implies that it is possible to capture the frequency information of incoming audio sound without FFT and new image shows a good potential to be alternative spectrogram-like visualized image for speech recognition application.

Because HT also may require significant computation burden similar to STFT, the different sound visualization method, recurrence plot (RP), is explored in this study. In general, the RP is a visualization of the recurrent states of dynamical systems^23^. Recurrence is a fundamental property of dynamical systems; it can be exploited to characterize the system’s behavior in phase space. In a wider context, recurrences have been recognized as a type of asymptotic invariant. The trajectory of any measure-preserving transformation must eventually return to the neighborhood of any former point with a probability of one. Along these lines, Eckmann et al. introduced RPs and the distance matrix in the late 1980s. This matrix contains a great deal of information about the underlying dynamical system and can be exploited to analyze the quantification of measured time series. For the speech recognition task, the raw acceleration signals are transformed into an image-like representation of recurrent states based on the distance matrices of recurrence plots^24^. CRPs were introduced as a bivariate extension of the RP designed to analyze the dependencies between two different systems by comparing their states based on nonlinear data analysis and have recently been widely adopted as a popular approach. The number and duration of recurrences of a dynamical system reconstructed by its phase space trajectory are quantified, and those variables can be used to characterize a dynamical system^25^.

**Figure 7:**
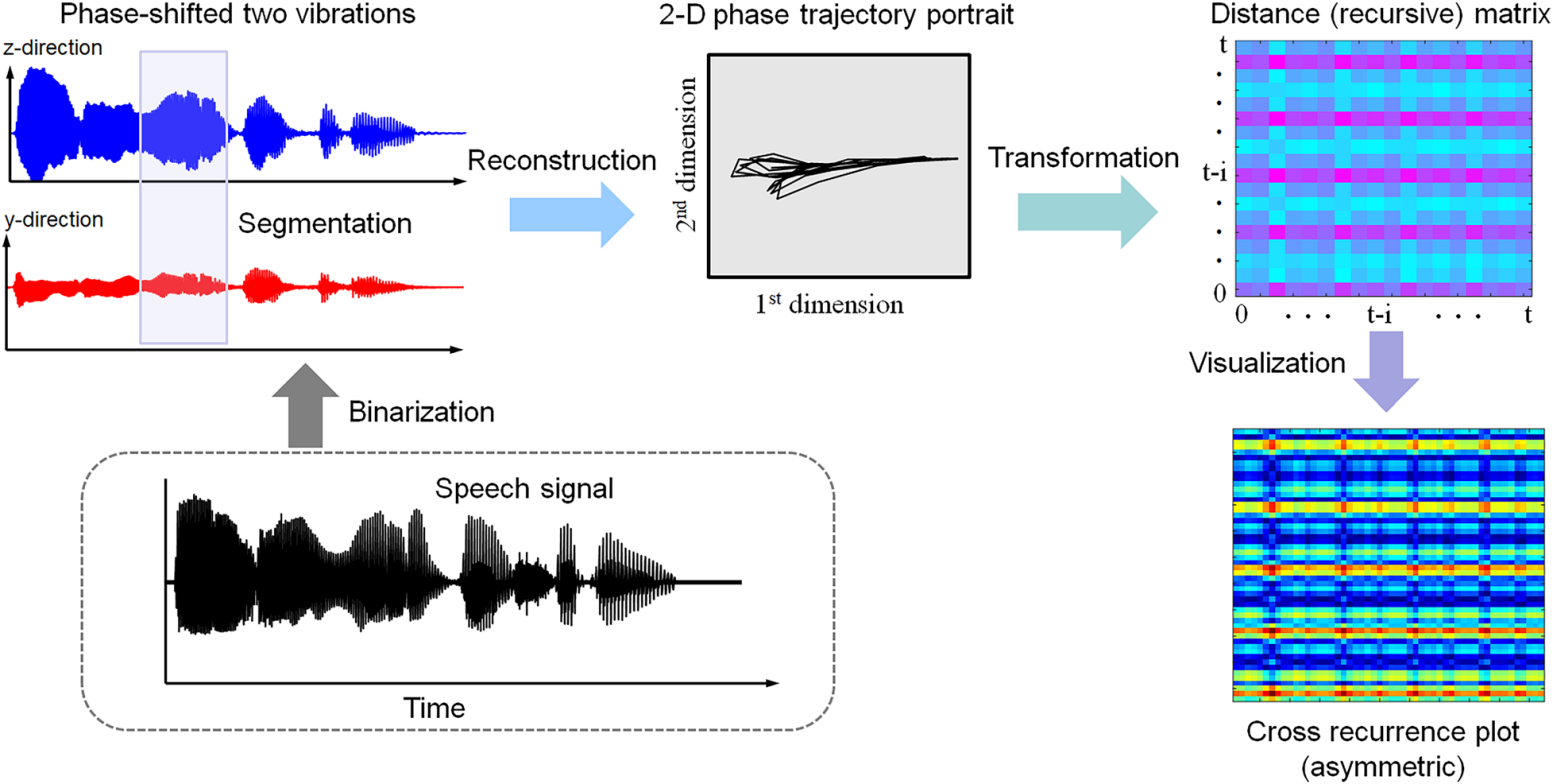
Input image generation framework based on CRP.

**Figure 8:**
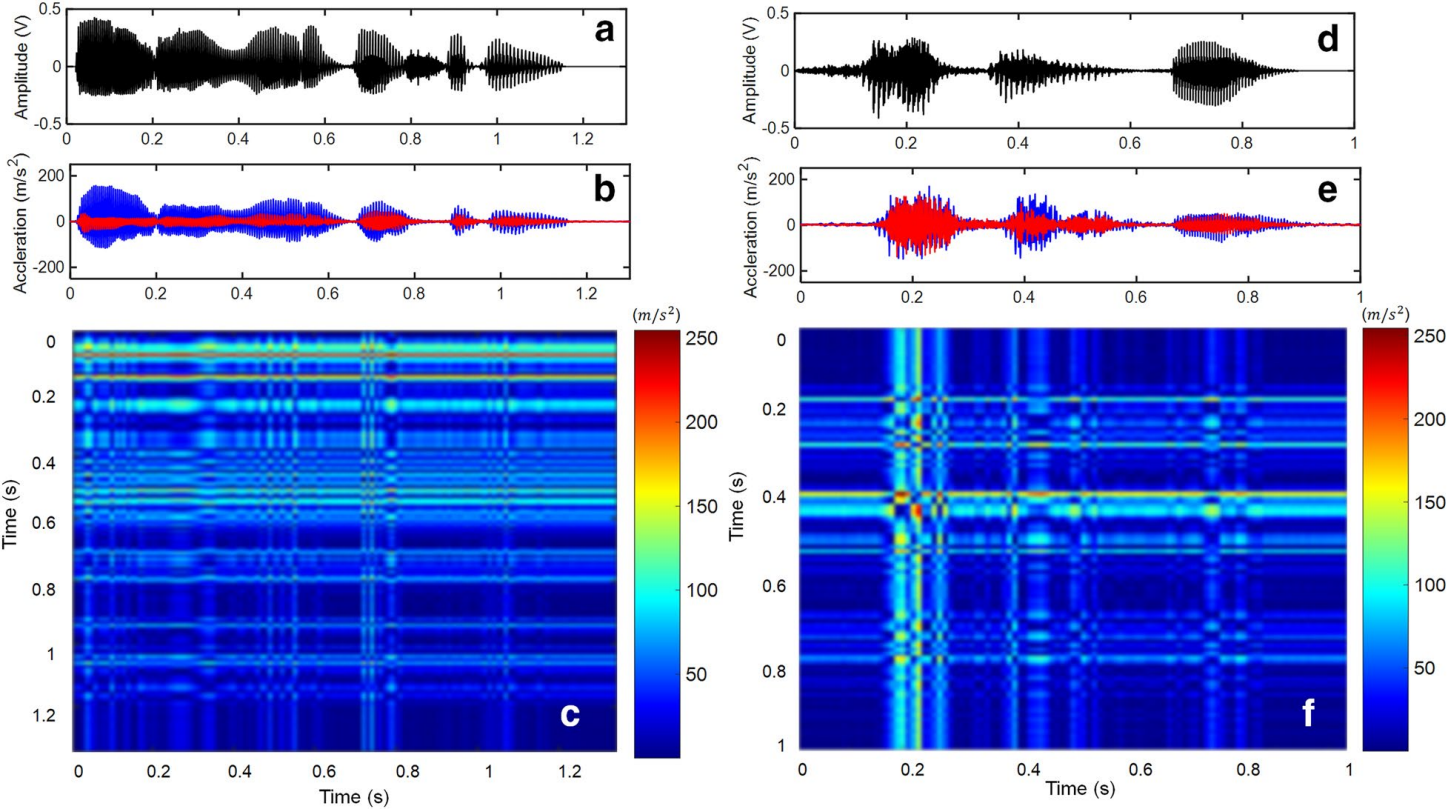
Cross-recurrence plot for speech signals. (**a**) speech signal of sound (Inha university), (**b**) its two acceleration signals, (**c**) corresponding CRP (pixel size: $134\; \times \;134$), (**d**) speech signal of the sound (happy house), (**e**) its two acceleration signals, (**f**) corresponding CRP (pixel size $134\; \times \;134$).

In this study, we transformed two phase-shifted time series into distance matrices of CRPs in a nonlinear manner. The overall framework for input image production based on CRP is shown in Fig. 7. The speech signal is first binarized into phase-shifted two vibrations using the new acoustic sensor with a viscoelastic diaphragm. Those two vibrations are segmented by a small window and reconstructed to a phase trajectory portrait on a 2D phase space. Then, phase trajectories are transformed into a distance matrix through simple computation. Finally, a CRP is visualized to colorize the distance matrices by those quantities. The details on CRP are described in the Methods section. The typical examples of input images formed by CRP are illustrated in Fig. 8. Two speech signals for the sentences, “Inha university” and “happy house” are used for test-bed speech signals. The later sentence is a compound of two words in “speech command test dataset,” which is available online^26^. The pixel size of 134 × 134 is used as the reference image resolution for the evaluation of computation time. Thus, the sampled input speech signal should be resampled to produce the pixel size of 134 × 134 (i.e., reconstruction).

**Figure 9:**
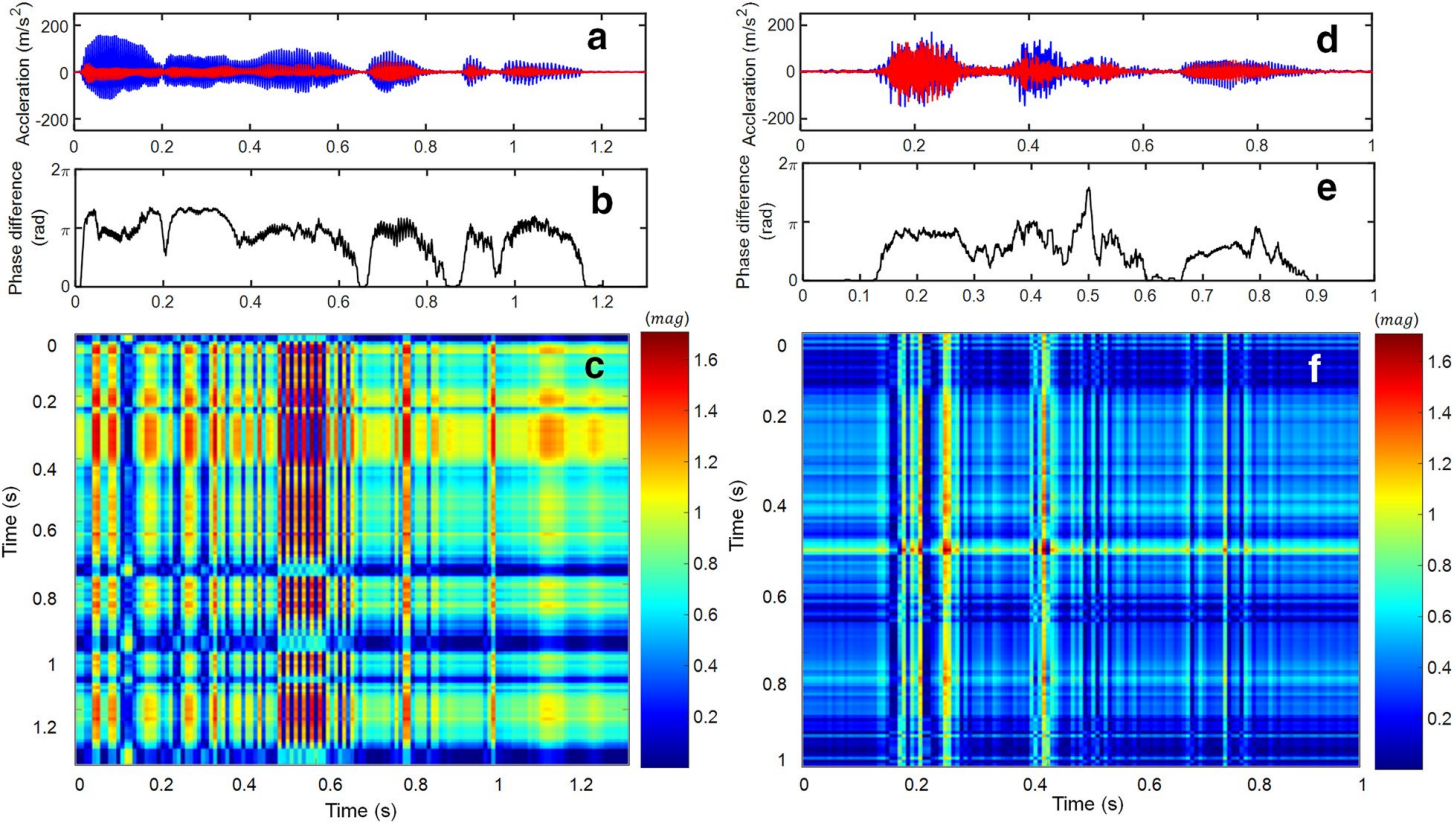
Cross-recurrence plot with HT for speech signals. (**a**) two acceleration signals of speech (Inha university), (**b**) their phase difference, (**c**) corresponding CRP (pixel size $134\; \times \;134$), (**d**) two acceleration signals of speech (happy house), (**e**) their phase difference, (**f**) corresponding CRP (pixel size $134\; \times \;134$).

Finally, the modified version of CRP is plotted by replacing the transverse vibration signal in the y-axis with the phase differences, as shown in Fig. 9. In the case of speech (Inha university), visualized 2D image becomes significantly different to the original CRP (i.e., Fig. 8c).

The CPU execution time required to produce the color image for speech (Inha University) recognition is compared in a simulation time of 1.2 s with a PC (CPU information: 11th Gen Intel^®^ Core™ i5-11400H), as summarized in Table 1. The CRP shows a lower computational burden. However, as the pixel size (resolution) increased, the CPU time of CRP increased and exceeded STFT when the pixel size is the critical size ($1138\; \times \;1138$) because the CRP requires the computation for the entire simulation time whereas the computation time (resolution) of STFT depends on the window size for each time instant. Overall, the CRP shows a lower computation burden and shows a promising potential alternative way to STFT (conventional spectrogram) when the image resolution (pixel size) is below critical resolution.

**Table 1:** Comparison of computation time.

Pixel size (resolution)	STFT (Fig. 6b) (s)	HT (Fig. 6e) (s)	CRP (Fig. 8c) (s)	CRP + HT (Fig. 9c) (s)
$134\; \times \;134$	0.150	0.158	0.005	0.167
$333\; \times \;333$	0.179	0.165	0.025	0.196
$666\; \times \;666$	0.230	0.174	0.102	0.280
$1138\; \times \;1138$	0.279	0.272	0.307	0.546

**Figure 10:**
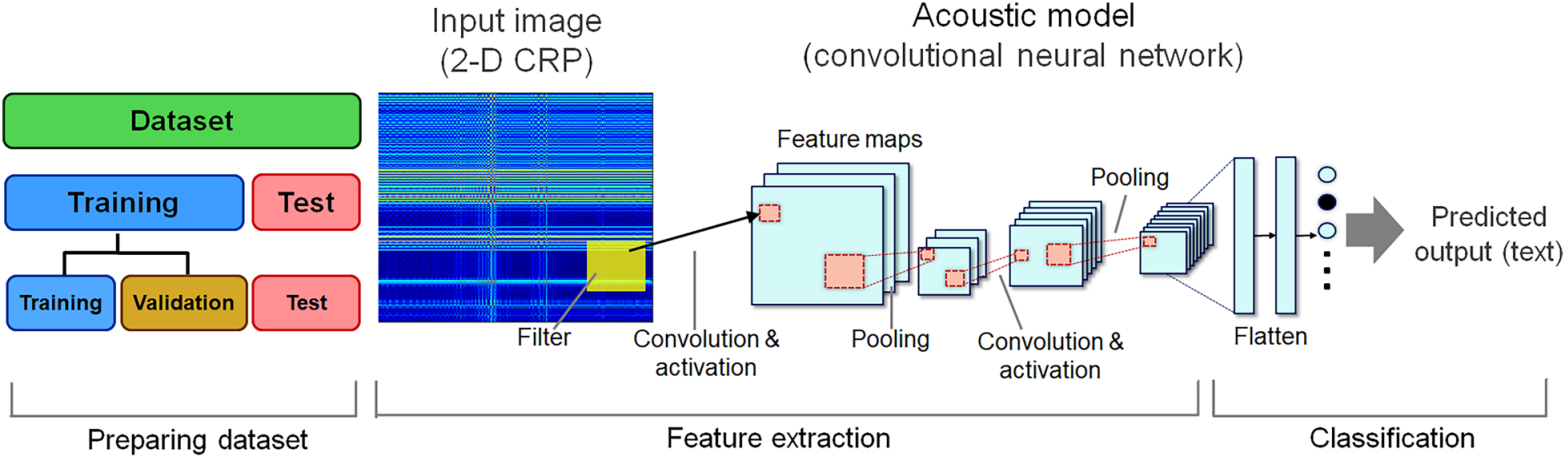
Architecture of the convolutional neural network model for speech recognition.

Finally, the CRP images can be used to train a CNN for real-time speech recognition^27^. The CRP image is $m \times n \times 3$ matrix, where $m \times n$ is width and height of image, and three is the number of color channels. Similar to spectrograms currently used in CNN-based speech recognition, CRP can be used for CNN model because its data is arranged by a continuous CNN-readable matrix of $m \times n \times 3$, which is the same as a typical spectrogram. Features of speech signals can then be extracted through convolution, pooling, and dropout, which refer to extracting speech features from filtered CRP images. The CRP image was asymmetric for entire periods whereas the conventional CRP for a single time series (original speech signal) was symmetric, which may be advantageous over conventional RP in terms of feature extraction. After extracting features, data are finally classified through a Softmax function, as shown in Fig. 10.

## Conclusions

In this study, we propose a new approach to speech recognition by producing different input images for a CNN model based on the two phase-shifted vibration responses produced by eardrum-inspired viscoelastic diaphragms. The proposed input images visualized by CRP for speech recognition can replace the current spectrogram formed by STFT. The CRP formed by two phase-shifted responses of viscoelastic diaphragm inspired by human TM (eardrum) can capture frequency information of incoming acoustic (audio, speech) signal. To the best of our knowledge, the present work is the first report the feasibility of new means of visualizing input sound. The main advantage of using the CRP is its lower computational resource requirements compared to a spectrogram, and it can be potential alternative means to STFT-based spectrogram if two phase-shifted responses can completely capture the frequency information of incoming sound (speech).

Overall, although this method of using viscoelastic responses offered promising applications for new speech recognition systems in terms of computational efficiency, there are some outstanding technical issues that remain to be addressed. For example, the robustness of the proposed method against noisy uncertainty should be further improved. Future studies may thus consider the improvement of the viscoelastic diaphragm with a primary focus on robustness, and the real implementation of the CNN-based speech recognition system with large training data sets. Future works should also seek a better understanding of the phase shift responses from viscoelastic diaphragms to improve the robustness of the proposed approach.

## Methods

The CRP is available in analyzing the signals modulated by a distance matrix, as shown in Fig. 7. The MATLAB Toolbox “Cross recurrence plot and quantification analysis -CRP, CRQA” was used to calculate distance matrices of two time series^28,29^. Time-series data of the two waves are defined as

5 $$\begin{gathered} x(t) = \vec{x}_{i} = \{ u_{1} ,u_{2} ,u_{3} , \cdot \cdot \cdot ,u_{n} \} \\ y(t) = \vec{y}_{j} = \{ v_{1} ,v_{2} ,v_{3} , \cdot \cdot \cdot ,v_{n} \} , \\ \end{gathered}$$

where $x(t)$ and $y(t)$ are time series, and $n$ is the order of the data. The natural numbers $i$ and $j$ indicate the $i{\text{th}}$ and $j{\text{th}}$ count, respectively. A threshold is applied to the distance matrix $CR_{ij}$ which isdefined as

6 $$CR_{ij} = \Theta (\varepsilon - \left\| {\vec{x}_{i} - \vec{y}_{j} } \right\|),$$

where $\Theta$ is the Heaviside-function, $\varepsilon$ is a specified threshold, and the thresholded distance matrix is transformed into diverse colors of pixels according to intensity and finally produces an image^28^. In this study, unthresholded CRP is produced for spectrogram-like training images for future application to deep learning algorithms for speech recognition. Extracting time series from two out-of-phase signals with a 1-ms time interval, CRP is shown in Fig. 8c,f, where the response in the z-direction is shown on the y-axis and that in the y-direction response on x-axis.

## Supplementary Information

Supplementary Figure S1.

## Data Availability

The datasets used and/or analyzed during the current study are available from the corresponding author upon reasonable request.
