# Supplementary figures and images for: Eardrum-inspired soft viscoelastic diaphragms for CNN-based speech recognition with audio visualization images
Source: Sci Rep. 2023 Apr 19;13:6414. doi: 10.1038/s41598-023-33755-2 (PMC10115895; doi:10.1038/s41598-023-33755-2)

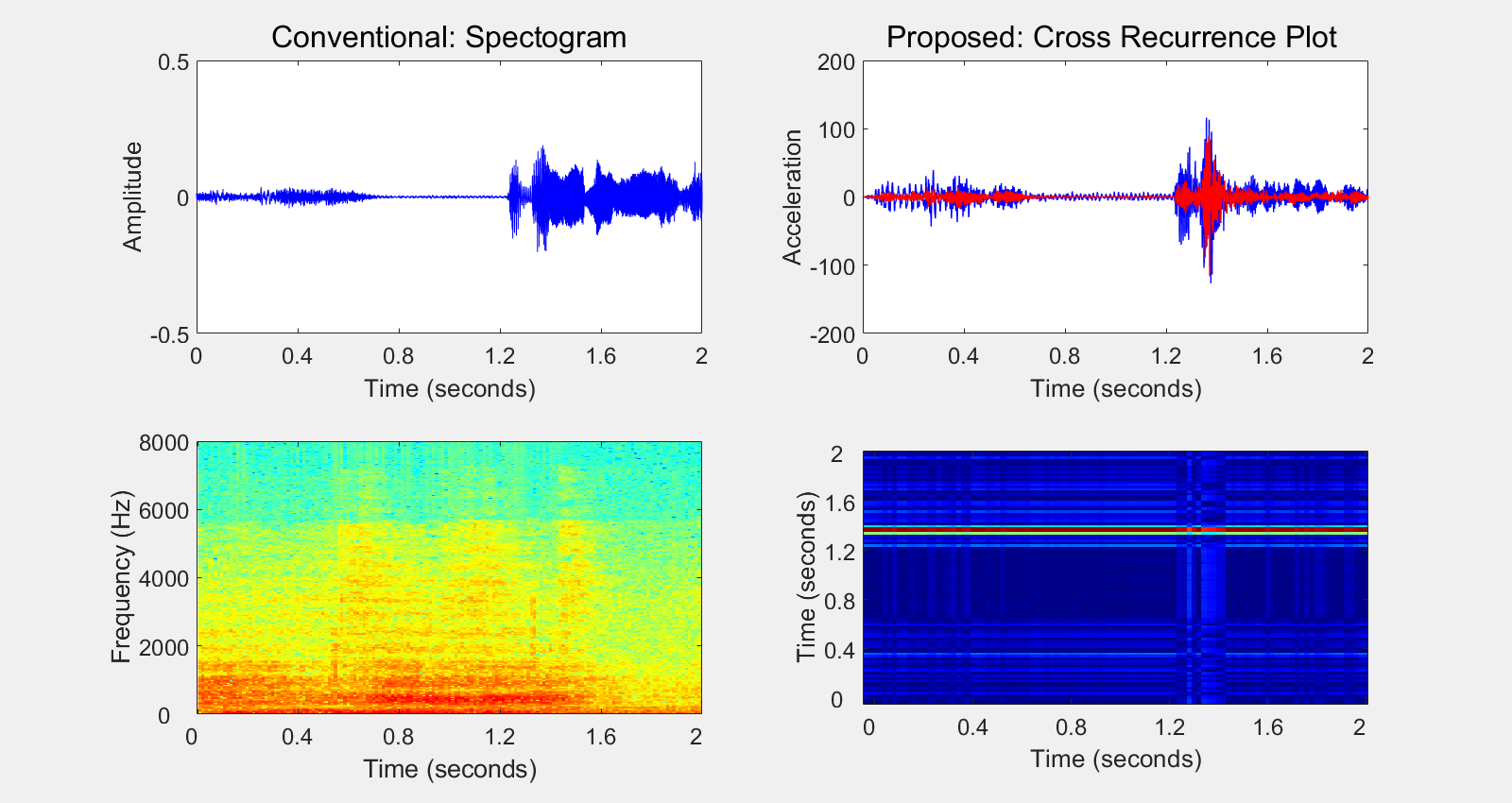

Supplement: Supplementary file 1 — Supplementary Figure S1. [file 41598_2023_33755_MOESM1_ESM.gif]
